# Supplementary material for: Differences in Mutational Profile between Follicular Thyroid Carcinoma and Follicular Thyroid Adenoma Identified Using Next Generation Sequencing
Source: Int J Mol Sci. 2019 Jun 26;20(13):3126. doi: 10.3390/ijms20133126 (PMC6651591; doi:10.3390/ijms20133126)
Supplement: Supplementary file 1 [file ijms-20-03126-s001.pdf]

| | Entity | AAE | Type | Alteration (physical location) | dbSNP or rs | COSMIC Mutation ID | Previously reported | Number of FTC patients with the mutation (n) | Number of FTA patients with the mutation (n) |
|---|---|---|---|---|---|---|---|---|---|
| *APC* | FTC | p.Thr1493 Thr | synonymous | chr5:112175 770 G>A | rs41115 | - | Familial colorectal cancer<br>Familial adenomatous polyposis 1<br>Hereditary cancer-predisposing syndrome<br>APC-Associated Polyposis Disorders | 4 | 1 |
| *CSF1R* | FTC FTA | - | 3 prime UTR variant | chr5:149433 596 T>G | rs206693 4 | - | Hereditary diffuse leukoencephalopathy with spheroids | 6 | 5 |
| *ERBB4* | FTC FTA | - | intron variant | chr2:212812 097 | rs839541 | - | - | 3 | 2 |
| *FGFR3* | FTC FTA | p.Thr653T hr | synonymous | chr4:180789 4 G>A | rs768860 9 | - | Craniosynostosis | 7 | 7 |
| *FLT3* | FTC FTA | c.1310-3T>C | splice region | chr13:28610 183 A>G | rs249123 1 | - | - | 18 | 10 |
| *FLT3* | FTC | - | intron variant | chr13:28602 292 | rs755808 65 | - | - | 5 | 0 |

| | | | | | | | | | |
|---|---|---|---|---|---|---|---|---|---|
| | | | | T>C | | | | | |
| *HRAS* | FTC FTA | p.His27His | synonymous | chr11:534242 A>G//T>C | rs12628 | COSM249860 | Costello syndrome Rasopathy | 6 | 7 |
| *KDR* | FTC FTA | - | intron variant | chr4:55980239 | rs7692791 | - | - | 3 | 2 |
| *PDGFRA* | FTC FTA | p.Val824Val | synonymous | chr4:55152040 C>T | rs2228230 | COSM22413 | Gastrointestinal stroma tumor; Idiopathic hypereosinophilic syndrome | 3 | 2 |
| *PDGFRA* | FTC FTA | p.Pro567Pro | synonymous | chr4:55141055 A>G | rs1873778 | - | Gastrointestinal stroma tumor; Idiopathic hypereosinophilic syndrome | 4 | 5 |
| *RET* | FTC FTA | p.Leu769Leu | synonymous | chr10:43613843 G>T | rs1800861 | - | Multiple endocrine neoplasia, type 2 | 11 | 10 |
| *RET* | FTC FTA | p.Ser904Ser | synonymous | chr10:43615633 C>G | rs1800863 | - | Pheochromocytoma; Renal adysplasia; Hereditary cancer-predisposing syndrome; Multiple endocrine neoplasia; Hirschsprung Disease, Dominant | 5 | 3 |
| *TP53* | FTC | p.Pro72Arg | missense | chr17:7579472 G>C | rs1042522 | - | Li-Fraumeni syndrome; Hereditary cancer-predisposing syndrome | 10 | 0 |
